# Supplementary material for: Association of lifetime e-cigarette and/or tobacco use and last year cannabis use among university students: A cross-sectional secondary analysis of a national survey
Source: PLOS Glob Public Health. 2022 May 25;2(5):e0000452. doi: 10.1371/journal.pgph.0000452 (PMC10022225; doi:10.1371/journal.pgph.0000452)
Supplement: Attachment — Submitted filename: Response to Reviewers .docx [file pgph.0000452.s003.docx]

January 25th 2022

'Response to Reviewers'.

Plos One Global Public Health

Dear Editor-in-Chief:

I wish to submit for publication in Plos One Global Public Health a revised version of the manuscript now titled “Lifetime e-cigarette and/or tobacco use and last year cannabis use among university students: a cross sectional secondary analysis of a National Survey” this paper was coauthored by Lucero Sangster-Carrasco and Dora Blitchtein.

The present document is the letter that includes the “Response to Reviewers” and that addresses each point raised during the review process by the editor and three reviewers,

Thank you for your consideration. I look forward to hearing from you.

Sincerely,

Coautors

Lucero Sanster-Carrasco	and	Dora Blitchtein*

*Correspondig author

Universidad Peruana de Ciencias Aplicadas]

Los Nogales 765 Apto 402 San Isidro. Lima, Perú.

+51 999090917

Dblit2007@gmail.com

Journal requirements

1. **The title in the manuscript file and the Title provided in your online submission form are the same**.

The actual revised title including reviewers’ suggestions is :

**“Lifetime e-cigarette and/or tobacco use and last year cannabis use among university students: a cross sectional secondary analysis of a National Survey” (lines 1-3)**

2. **Please provide separate figure files in .tif or .eps format only and ensure that all files are under our size limit of 20MB. For more information about how to convert your figure files please see our guidelines:** <https://journals.plos.org/globalpublichealth/s/figures>

Our only figure: Figure 1 Study participants selection flowchart. Was saved in tiff format and the size is 3.1 MB.

3. **Please amend your detailed Financial Disclosure statement. If you did not receive any funding for this study, please simply state: “The authors received no specific funding for this work.”**

The information about Financial Disclosure statement has been updated with the following information:

“The authors received no specific funding for this work. (Line 433)

4. **Please update the completed 'Competing Interests' statement. If you have no competing interests to declare, please state: “The authors have declared that no competing interests exist.”**

The information about 'Competing Interests' statement has been updated with the following information:

“The authors have declared that no competing interests exist. (line 430)

5. **In the online submission form, you indicated that “The database of this study can be accessed through the request an acceptance of Office of United Nations Against Drug (UNODC) and the Comisión Nacional para el Desarrollo y Vida sin Drogas (DEVIDA) from Peru.”. All PLOS journals now require all data underlying the findings described in their manuscript to be freely available to other researchers, either 1. In a public repository, 2. Within the manuscript itself, or 3. Uploaded as supplementary information.
   This policy applies to all data except where public deposition would breach compliance with the protocol approved by your research ethics board. If your data cannot be made publicly available for ethical or legal reasons (e.g., public availability would compromise patient privacy), please explain your reasons by return email and your exemption request will be escalated to the editor for approval. Your exemption request will be handled independently and will not hold up the peer review process, but will need to be resolved should your manuscript be accepted for publication. One of the Editorial team will then be in touch if there are any issues.**

We will upload as supplementary information the database with variables and population of our study.

6. **All main tables must be in an editable format for typesetting. Please include Tables 1, 2 and 3 in your main text as an editable table and not an image.**

Tables 1, 2A, 2B and 3 are insert in editable excel object format in the main manuscript document.

7. Review Comments to the Author

**Please use the space provided to explain your answers to the questions above. You may also include additional comments for the author, including concerns about dual publication, research ethics, or publication ethics. (Please upload your review as an attachment if it exceeds 20,000 characters)**

**Reviewer #1: E-cigarette and/or tobacco use and cannabis use among university students
This an interesting which assess risk factors for NCD in southern America. It is very stricking there few studies on these risk factors and uthours have conducted a cross-sectional study amaongs younger population. It will have been more powerful is this covered adult and older population as a population-based survey.**

There few minor points.

**a) It is important to clearly emphasise the nature and design of survey - which is cross sectional. Importantly cross-sectional survey only provide point prevalence rather than over time (longitudinal).**

**Suggestion accepted**

In the title we included the cross sectional study design, also in the abstract and in methods section was already stated (line 81-83)

Title change from : “E-cigarette and/or tobacco use and cannabis use among university students”

to

“Lifetime e-cigarette and/or tobacco use and last year cannabis use among university students: a cross sectional secondary analysis of a National survey.” (lines 1-3)

It was also already included in Abstact, Material and Methods and Discussion sections of the manuscript :

In Abstract section : “Design: An analytical cross-sectional study was conducted based on the III Andean epidemiological study on drug consumption among university students in 2016” (lines 26-29),

Material and methods section “This was a cross-sectional analytical study secondary based on the III Andean epidemiological study on drug use in the university population of Peru in 2016” (lines 150-152),

and in limitations in the Discussion section “The study has some limitations as well, such as being a secondary analytical cross-sectional study and a direct causal relationship cannot be attributed since it does not include progression over time..”(lines 389-391)

**b) statistical comparisons are fine - but i found some lack of clarity on how many use tobacco and proportion of those that use cannabis from the survey. Further how this proportion compares to other studies. This needs further re-reading and refinement in write up.**

**Suggestion accepted**

In results section we included the lifetime and past year prevalence use of tobacco and cannabis and the lifetime prevalence of e-cigarette use in this population, we couldn’t compute the annual e-cigarette use prevalence because in the original study they didn’t assess about last year e-cigarette use.

“Regarding cannabis use, 14.8% (CI 95% 13.0 to 16.7%) reported having used cannabis in their lifetime; the prevalence of use in the last 12 months was 5.2% (CI 95% 4.1 to 6.5%).,while regarding tobacco use, 53.1% (CI 95% 50.5 to 55.7%) reported having used tobacco in their lifetime: the prevalence of use in the last 12 months was 29.6% (CI 95% 27.3 to 32.1%), regarding e-cigarettes 12.7% (CI 95% 11.2 to 14.5%) reported having used e-cigarettes in their lifetime.” (lines 245-250)

Also in the Discussion section we included how the most relevant proportions aligned with the aim of this study compared to other studies

“The lifetime prevalence of dual use of e-cigarettes and tobacco in Peruvian university students was 11.6% (CI95% 10.1 ;13.3) ; only tobacco was 41.5% (CI95% 38.9 ;44.2); neither tobacco nor e-cigarettes was 45.7% (CI95% 43.1 ;48.4); and only e-cigarettes was 1.2% (CI95% 0.7 ;1.8). These results in the university population contradict the study on high school students in the United States, where one study found that the highest prevalence was reported in the use of e-cigarettes only (18%), when compared to tobacco (only) use (3%), and dual use of e-cigarettes and tobacco (13%).[29] These differences could be because e-cigarettes are perceived as healthier than tobacco,[37] and due to the varying regulations and access to e-cigarettes, tobacco, and cannabis in each country.[6] Additionally, ethnic and/or cultural patterns could have an influence.[35]” (lines 375 – 384)

**c) it is better to rely on point prevalence and 95% CI e.g. Wald. something authours can easily extract from Stata or R. Example on Table 2. P-value fine but do not offer any further infomation on uncertainty of estimate or how wide CI is. Same applies to reporting in text.**

**Suggestion accepted**

As shown in Tables 1, 2A, 2B bellow, 95% confidence intervals were computed and included,:

Table 1 with 95% confidence intervals

Table 2A with 95% confidence intervals

Table 2B with 95% confidence intervals

And in the descriptive results section test we included 95% CI of substance use point prevalence values

“Regarding cannabis use, 14.8% (CI 95% 13.0 to 16.7%) reported having used cannabis in their lifetime; the prevalence of use in the last 12 months was 5.2% (CI 95% 4.1 to 6.5%).,while regarding tobacco use, 53.1% (CI 95% 50.5 to 55.7%) reported having used tobacco in their lifetime: the prevalence of use in the last 12 months was 29.6% (CI 95% 27.3 to 32.1%), regarding e-cigarettes 12.7% (CI 95% 11.2 to 14.5%) reported having used e-cigarettes in their lifetime.” (Lines 245-250)

**d) What don’t we know from this study? I find the proportion rather small in terms of those who use tobacco and go on to use Cannabis although the odds are strikingly large. It is obvious that cannabis users would have smoked tobacco at some stage. I think the manuscript would be improved by drawing out the overall risk behavior, NCDs and effects of Tobacco use in this population. Because we know little in terms of NCD burden, risk factors. What role could routinely collect data help improve further studies?**

**This comment was answered in for different sections: d1,d2,d3,d4, containing different suggestions:**

**d.1) I find the proportion rather small in terms of those who use tobacco and go on to use Cannabis although the odds are strickingly large.**

**Comment answered bellow**

About the comment “**I find the proportion rather small in terms of those who use tobacco and go on to use Cannabis although the odds are strikingly large”** .Is the same relation found in other studies [Azagba, 2018 ) only it seems larger because we measure lifetime use of tobacco and/or e-cigarette and last year cannabis use.

**d.2) It is obvious that cannabis users would have smoked tobacco at some stage.**

**Suggestion included (Theories of explanation of the relation between nicotine and cannabis)**

Regarding to the comment “**It is obvious that cannabis users would have smoked tobacco at some stage”** we included the theories that explain the relation between nicotine containing products and cannabis in the introduction

“The relation between nicotine and cannabis use can be explained trough different theories, the “Common Liability Theory”, supports that there are genetic and or environmental factors that influence a greater risk of use, and abuse or dependence of either nicotine or cannabis .While the “Route of Administration Theory” explains this relation on the fact that both, nicotine and cannabis, are mostly used trough the same route of administration, smoked or vaporized, this route has more rapid and lasting effects, the underlying mechanisms after the initiation of substance use are : respiratory adaptation, positive expectations and favorable perception favors use of other substances by the same route. While according to the Gateway theory there is a predictable progression in drug use, beginning with legal, followed by illegal drug use. This last theory is adapted to the characteristics of the consumption of psychoactive substances in the country. (line 120-129)

**d.3) I think the manuscript would be improved by drawing out the overall risk behaviour, NCDs and effects of Tobacco use in this population.**

**Suggestion already included, and we added bibliography .**

In reference of the “**improvement of the manuscript by drawing out the overall risk behavior”**, it was included in the manuscript paragraph :

“This increase in young people’s usage of e-cigarettes, is: due to its novelty; the perception that it is socially acceptable; the positive expectations it generates, such as gaining respect, improving the possibility of being liked by others, and being popular; its stress-relieving effects; boredom; and sensory experiences, such as enjoying smell and taste.[2, 7] Additionally, they believe: e-cigarettes are not harmful or are less harmful than the common tobacco cigarettes; they have pleasant smells and tastes; they are useful to stop smoking; and there are no restrictions on use in public places where tobacco smoking is not allowed. Most adolescents and young people are not aware that nicotine levels can be very high in these products,[8]” (lines 85-94)

And we included additional bibliographic references of identified risks for e cigarette and dual use in the included variables references. (lines 177-181 and 218-220)

**d4)** In reference to the comment about **“drawing out the overall risk behavior, NCDs and effects of tobacco use in this population”**

**Suggestion partially included	(limitations in Discussion section)**

The suggestion exceeds the aim of the present study that measured tobacco and or e-cigarettes association with cannabis, the analysis of NCD and Tobacco effects deserves a specific study, there is a recent longitudinal study about this topic [Staff 2022].

Staff J, Kelly BC, Maggs JL, Vuolo M. Adolescent electronic cigarette use and tobacco smoking in the Millennium Cohort Study. Addiction. 2022;117(2):484-94.

With the actual study, a cross-sectional design and without some more specific information about e-cigarettes that wasn’t included in the original study we can’t measure risks of effects or progression from NCD to tobacco or tobacco to NCD.

We included in limitations section this gap of information:

“The study has some limitations as well, such as being a secondary analytical cross-sectional study and a direct causal relationship cannot be attributed since it does not include progression over time, only association…………. Also, no information was collected about the sequence of use of e-cigarettes and/or tobacco or whether they are consumed simultaneously, the component or levels of nicotine or cannabis products used as inputs in e-cigarettes were not specified, nor if one type was used exclusively or if they could be used alternately. “(Lines 389-400)

**Reviewer #2:**

1. **Abstract is poorly written. Please elaborate your objectives with background why you want to investigate this issue.**

**\**

**Suggestion accepted**

The background section has been added with an elaboration of the aims of the study as suggested, the content of the abstract has been reviewed as it is shown below. (lines **16-51**)

2. **.All minor sections in the Abstract to be added in one sub-heading Methods**.

**Suggestion accepted**

**The minor sections were grouped into a single Methods section in the abstract , as shown below (lines 25-33)**

**“Background**

In recent years, the emergence and rapid increase of e-cigarette use has been added to the usual route of nicotine consumption through smoked tobacco, expanding the possible options of patterns of lifetime nicotine use to only tobacco, electronic cigarette only, dual use or neither, and opened a full range of possibilities in the dynamic of nicotine use, including dose, simultaneous use and frequency, the relation between lifetime patterns of nicotine use and cannabis use has yet to be determined. The present study examined the association between the lifetime use of electronic cigarettes and/or tobacco, and the use of cannabis in the last 12 months.

**Methods**

**Design**: An analytical cross-sectional study was conducted based on the III Andean epidemiological study on drug consumption among university students in 2016. A multivariate analysis was performed using a generalized linear model of the family and the log Poisson link option. **Setting**: Ten Peruvian universities. **Participants**: University students **Exposure**: Participants’ e-cigarette and/or tobacco consumption, based on questions about lifetime consumption of tobacco and e-cigarettes. The participants were categorized into those consuming: only e-cigarettes, only tobacco, both, or neither. **Outcome**: Cannabis use by participants in the last 12 months

**Results**: Among the university students who met the inclusion criteria (n=3981), the prevalence of use of e-cigarettes and tobacco (dual) throughout life was 11.6% (CI 95% 10.1 to 13.3), and only e-cigarettes was 1.2% (CI 95% 0.7 to 1.8). The use of cannabis in the last 12 months was 5.2% (CI 95% 4.1 to 6.5). Results of this study found a sequential probability gradient of cannabis use, were those with dual use were almost 58.5 times more likely to have used cannabis in the last 12 months (PR 58.5, CI95: 20.9 to 163.7, p <0.001) compared to those who used none; whereas those who used only tobacco were 33.3 times more likely to have used cannabis in the last 12 months (PR 33.3, CI95: 11.9 to 93.2, p <0.001), while among those who reported only e-cigarette use, were 9.8 times more likely to have used cannabis in the last 12 months (PRa 9.8, 95% CI 1.6 to 60.4, p = 0.014).

**Conclusions**: We found an increase in the probability of cannabis use in the last 12 months among university students who reported tobacco and /or e-cigarette use. A sequential probability gradient was found in which lifetime dual users university students were most likely to use cannabis in the last 12 months, followed by those who reported lifetime exclusive use of tobacco and those who reported lifetime exclusive e-cigarettes use compared with those students who reported being nonusers of tobacco and/or e-cigarette. “

**3. Title is very confusing: in fact you aimed to study determinants of cannabis use in the last 12 month and those using e-cigarettes and tobacco consumption have very high risk factor of using cannabis.This should reflect in the title.**

**Suggestion accepted**

Title change from : “E-cigarette and/or tobacco use and cannabis use among university students”

to

“Lifetime e-cigarette and/or tobacco use and last year cannabis use among university students: a cross sectional secondary analysis of a National survey.” (lines 1-3)

4. **All tables have too much information on demographic and socioeconomic and behavioral characteristics of students**.

**Suggestion partially accepted**

Based on yours and other reviewers’ suggestions we reduced some of the sociodemographic information in tables 1, 2A and 2B, some of the variables were related to younger cohorts in other studies, therefore we decided to exclude father and mother relation with the university student and parents’ marital status.

Additionally, as part of other reviewers’ suggestions, we included the age of initiation of cannabis use, availability of cannabis in last 12 months and cannabis exposure opportunity in last 12 moths, we also reviewed and simplified other variables.

**5. Table 2A is misleading by providing life-time prevalence/use of tobacco, etc. Authors should have focused on current or past 12 month prevalence rate.**

**Suggestion partially accepted**

The exposure variable was lifetime tobacco and or e-cigarette use. Therefore, in **table 2 B** the timeframe if lifetime for tobacco and or e-cigarette.

In **Table 2A** we reviewed time frame and we focus in past 12 months for most of the variables, also in the **Results section**, as shown below, in text we included life and past year prevalence of tobacco and cannabis use, and lifetime e cigarette use. (line 245-250)

“Regarding cannabis use, 14.8% (CI 95% 13.0 to 16.7%) reported having used cannabis in their lifetime; the prevalence of use in the last 12 months was 5.2% (CI 95% 4.1 to 6.5%).,while regarding tobacco use, 53.1% (CI 95% 50.5 to 55.7%) reported having used tobacco in their lifetime: the prevalence of use in the last 12 months was 29.6% (CI 95% 27.3 to 32.1%), regarding e-cigarettes 12.7% (CI 95% 11.2 to 14.5%) reported having used e-cigarettes in their lifetime.”

6. **The findings are obvious that those who have smoking experience have used cannabis. No body i.e. a non-smoker will not straight away jump into cannabis smoking.**

**Comment answer**

Even if the results shown risk between all patterns of lifetime nicotine use(tobacco or e-cigarette), there was a gradient of cannabis use probability, the highest probability compared with the university students who declared non use of either, the highest was Tobacco & e-cigarette use followed by tobacco only and exclusive e-cigarette use, more longitudinal research should be perform to inquire about progression to cannabis use from one to the other, simultaneous use, nicotine dose, and cannabis vaping.

Regarding to the comment . “**No body i.e. a non-smoker will not straight away jump into cannabis smoking.”** we included the theories that explain the relation between nicotine containing products and cannabis in the introduction

**Suggestion included (Theories of explanation of the relation between nicotine and cannabis)**

“The relation between nicotine and cannabis use can be explained trough different theories, the “Common Liability Theory”, supports that there are genetic and or environmental factors that influence a greater risk of use, and abuse or dependence of either of nicotine or cannabis .While the “Route of Administration Theory” explains this relation on the fact that both, nicotine and cannabis, are mostly used trough the same route of administration, smoked or vaporized, this route has more rapid and lasting effects, the underlying mechanisms after the initiation substance use : respiratory adaptation, positive expectations and favorable perception favors the use of the other substance by the same route. While according to the Gateway theory there is a predictable progression in drug use, beginning with legal, followed by illegal drug use. This last theory is adapted to the characteristics of the consumption of psychoactive substances in the country. (line 120-129)

**7. In statistical section authors have not provided any justification of choosing GLM over Logistic Regression model.**

**Suggestion accepted**

We included in the methodology section the argument and bibliographic reference for using GLM over logistic regression and PR instead of OR (line 293 -299)

For the crude and adjusted multivariate analysis, a generalized linear model (GLM) and the log Poisson link option was applied, allowing precise model building and providing statistical measures to assess its fit [Dias 2013], measure of association were presented as prevalence ratio (PR),this measure was selected over odds ratio (OR) and Logistic Regression commonly used in drug use surveys analysis, because is the interpretation is easier and prevents overestimation. [Espelt 2017] [Tamhane 2016][Dohoo 2009]

8. **Too many covariates/confounders are used without providing any logical reasoning or in conceptual framework for inclusion**.

**Suggestion accepted**

We reviewed all the suggestions including only confounders identified in other related studies, using an epidemiological criterion to include them in the adjusted model there are: age, socioeconomic level, sex, alcohol use in las 12 months and illegal drug use, references are cited in the statistical methods section (lines 217-219)

An epidemiological criterion was considered for entry of the variables in the adjusted models, which were age category, sex,[18] socioeconomic stratum,[32] alcohol consumption in the last 12 months,[15] and illicit drug use.[15]

**9. Discussion section lacks reasoning about pivotal role these confounding variables have played**.

**Suggestion already included and improved with recent modifications**

The discussion section has been updated and the findings of the study are presented in context, including the role of some key variables included in the final adjusted model, as well as others identified in the literature. (lines 305-399)

Reviewer #3:

Please see my document attached with an account of what I consider should be tackled in order to improve our knowledge regarding gateway theory including a different method to approach the association between e-cigarette and cannabis.

# E-cigarette and/or tobacco use and cannabis use among university students. Dear authors

**I am extremely sympathetic to this important research, particularly because I believe knowing more about other countries rather than confirming or rejecting research in North America or Europe is very necessary. Having said that I highly recommend clarifying methodological issues if authors pursue with the idea of moving forward the “gateway” theory, and therefore model the survey with at least two considerations I describe below. If on the other hand authors drop the gateway theory, this paper could be very well a descriptive one which is also very fine.**

**Introduction**

1. **Authors reflect on a high prevalence rate of e-cigarette, in Peru lines 63-67, but this conclusion is first redundant (lines 63 and 64 mean the same as lines 65-67) and second, the novelty of these devices may not be actually reaching any saturation yet. Your research indicates to poles Italy and Poland, yet Peru is a lot closer to Italy assuming the research you cite is representative of Peru.**

**Suggestion about redundance in lines 63-67, and lines and lines 65-67) included**

We synthetized duplicated information. (lines 81-82)

2. **You mentioned multiple reasons lines 68-77, I would then avoid any reference to lack of policies because it is still too early to determine its “missing” impact. Also these policies take years in showing effects.**

**Suggestion accepted**

In the previous paragraph (81,82) we excluded the concept of exclusive legal norms effects, now it refers to general rules surrounding e-cigarettes use, including all kind of regulations (ie. social,marketing, and others)

3. In lines 88-89 I don’t observe a “significant” increase in women prevalence. The 1% increase in this population should be tested, if this is the case please at least refer to the confidence intervals for each comparing year to identify such change. By contrast consumption in men is likely much higher given the differences.

**Suggestion accepted**

We reviewed the prevalence information from the reference article and the paragraph was modified, only to include the significant increase in men, as follows :

“However, in Latin American countries, the most consumed illicit substance by the youth population is cannabis. In Peru, the prevalence of cannabis use among university students increased significantly between 2009 and 2012: from 10.5% to 16.9% in men, and **not significatively,** from 5.6% to 6.6% **in women**. Simultaneously, there was a fivefold increase in annual incidence in Peru, from 0.48% (2009) to 2.78% (2012).[12] The increase in cannabis use among university students in Latin America may be associated with changes in the perception of risk.” (line 103 – 107)

4. **The literature on changes on cannabis use is extremely large. Authors do not provide any reference (lines 90-92) to suggest that perception of risk is one leading factor to understand this change in Latin America. Please bring a more complete picture of this change. I believe work by the Pan American Health Organization could be a good reference.**

**Suggestion partially accepted**

This role of risk perception and cannabis increased use in Peru and Latin America has already been described, we included the correspondent bibliographic reference: [Hynes 2015]	(line 109)

**.**

5. **There is no need to introduce literature on effects or consequences of cannabis use in this manuscript unless researchers are testing consequences of this substance.**

**Suggestion not accepted**

We consider that cannabis use consequences are relevant for this study and Public health considerations arising from its results, we will keep them in the manuscript.

6. **If authors would like to “apply” the gateway theory, then they should introduce other theories that explain this transition at all (i.e. “common liability theory (to engage in risky behavior)”). However, a methodological problem remains which is the use of a cross sectional survey to identify changes over time.**

**Suggestion included (Theories of explanation of the relation between nicotine and cannabis)**

We included this valuable suggestion in paragraph (line 120-129)

“The relation between nicotine and cannabis use can be explained trough different theories, the “Common Liability Theory”, supports that there are genetic and or environmental factors that influence a greater risk of use, and abuse or dependence of either of nicotine or cannabis .While the “Route of Administration Theory” explains this relation on the fact that both, nicotine and cannabis, are mostly used trough the same route of administration, smoked or vaporized, this route has more rapid and lasting effects, the underlying mechanisms after the initiation substance use : respiratory adaptation, positive expectations and favorable perception favors the use of the other substance by the same route. While according to the Gateway theory there is a predictable progression in drug use, beginning with legal, followed by illegal drug use. This last theory is adapted to the characteristics of the consumption of psychoactive substances in the country.

# Methods.

**Authors should model, if they are interested in “testing” the gateway theory, results using structural equation models to observe how “over time” the dependent variable is associated with the independent variables. One extreme limitation of the study is the independent variable which considers “in your life” sentence; this could mean actually “after” any consumption of cannabis. I suggest instead at least consider date in which individuals declare smoke e-cigarette for the first time and comparing to question first time individual consume cannabis for the first time. This will correct partially for the time concern that is associated with the theory authors suggest they use. Among the controls authors should at least consider access to cannabis following questions: “Cuando fue la ultima vez que le ofrecieron algunas de estas drogas, ya sea para comprar o probar?” “¿Cuán fácil o difícil le sería conseguir alguna de las siguientes drogas?”**

**Suggestion accepted and all possible actions has been taken**.

Thanks for the valuable suggestion, it was not possible to include the date in which individuals declared smoke e-cigarette because it wasn’t included in the original study.

We included the age of cannabis initiation (mean and SD) in tables 1, 2A in the results section, and in the same tables the variables about last year cannabis availability and cannabis exposure opportunity, as was suggested, as it is shown in next pages

Table 1

Table 2A

1. **Authors should introduce literature that suggest why they are using all the control variables suggested in lines 152-154.**

**Suggestion accepted**

We reviewed all the suggestions including only confounders identified in other related studies, using an epidemiological criteria to include them in the adjusted model there are: age, socioeconomic level, sex, alcohol use in las 12 months and illegal drug use, references are cited in the statistical methods section (lines 217-219)

An epidemiological criterion was considered for entry of the variables in the adjusted models, which were age category, sex,[18] socioeconomic stratum,[32] alcohol consumption in the last 12 months,[15] and illicit drug use.[15]

2. **Problems with the modeling chosen and results.**

**2 a.- Work in addition to studying is probably correlated with socioeconomic status.**

**Suggestion accepted**

We included your suggestion and computed a new adjusted model without “work in addition to studying”and without “parental control” as was suggested by other reviewer as shown in Table 3

**There is a problem with the tables because in table 2A the n of e- cigarettes is 62 and in table 2B e-cigarette is 108. This is a huge discrepancy. If on the other hand this second number correspond to e-cigarette use AND tobacco then the discrepancy still remains because 478+108=586 and in table 2A this corresponds to 524.**

**Suggestion accepted and solved .**

Table 2 A

This observation was review and solved in Table 2A and 2B, and confidence intervals 95% were included also.

Table 2 B

3. **There is more than 10% of discrepancy that needs to be clarified.**

**Suggestion accepted and solved in Table 2A and 2B**

4. **Table 3 does not have information of how many individuals are considered in the analysis.**

**Suggestion included,** on the left side of Table 3 Title the number was included (n=3981)

5. **(Important recommendation: authors should definitely model an interaction effect with age to understand how “over time” a cohort effect could be observed. One could thus assume that e-cigarettes have been less prevalent and therefore younger cohorts could be more exposed to e-cigarette and then maybe observing a change in cannabis consumption.**

**Suggestion accepted and all possible actions has been taken**.

We included the mean and SD of age of initiation of cannabis use according of e-cigarette and or tobacco use categories in Table 2A and this result suggest that e-cigarettes users only initiated it use earlier than other categories.

There is no information about age of initiation of e-cigarettes and we can’t perform the full structural equation model without this information.

Although we did our best efforts to include your suggestions in a sub analysis, we didn’t have enough population size of age cohorts for a confident analysis of interaction effect. And information about age of initiation of e-cigarettes was not available for the structural equation model suggestion.

We included in limitations the absence of information of the age at first use of e-cigarette

In STATA 17 MP gsem (e_cigar_n -> EDADINI_MAR, family(gaussian) link(identity)) (tobacco_life -> EDADINI_MAR, family(gaussian) link(identity)), nocapslatent

# Discussion

**The interpretation of the gateway theory should be avoided since as I mentioned no time control is provided. It is not sufficient to mention in the limitations that is a cross sectional study, this is also why I suggest doing both a structural equation model and interaction effects with different cohorts. So, if authors would like to proceed with this theory, then they should examine if cannabis changes for the cohort of age 25 its prevalence of first time cannabis use is likely different than the youngest cohort. With the gateway theory applied to e-cigarettes and cannabis consumption one could observe that cannabis consumption should be higher in the youngest cohort relative to the oldest one because e-cigarettes explain more variance after its increment. If on the other hand, first time cannabis consumption in both cohorts are not different from each other then no evidence of e-cigarette has been identified; which by the way is more than fine. Last, there is inherent desirable bias when answering drugs surveys, this should be acknowledged in the limitations.**

Although we did our best efforts to include your suggestions in a sub analysis, we didn’t have enough population size of age cohorts for a confident analysis of interaction effect. And information about age of initiation of e-cigarettes was not available for the structural equation model suggestion.

With the information about younger age of onset of cannabis use by e-cigarettes only users, based on your suggestions, we included in the Discussion the suggestion of further cross-sectional studies with enough information and or longitudinal studies to assess progression from nicotine to cannabis.

We included in limitations the absence of information of the age at first use of e-cigarette and also the inherent recall and desirability bias included in self-report drug surveys.

In the adjusted model younger age was related with more probability of cannabis use in last 12 months, al least 3 times more in cohorts 18, 19-20,21-22, compared with university students older than 25 years old group, the reported age of onset of use of cannabis was younger in 18, 19-20 and 20-21 cohorts

The mean age of cannabis first use in age cohorts was younger in earlier cohorts.
