# Supplementary material for: Association of lifetime e-cigarette and/or tobacco use and last year cannabis use among university students: A cross-sectional secondary analysis of a national survey
Source: PLOS Glob Public Health. 2022 May 25;2(5):e0000452. doi: 10.1371/journal.pgph.0000452 (PMC10022225; doi:10.1371/journal.pgph.0000452)
Supplement: Attachment — Submitted filename: Point by point answers to reviewers 26th march.docx [file pgph.0000452.s004.docx]

'Response to Reviewers'.

Plos One Global Public Health

Dear Editor-in-Chief:

We wish to submit for publication in Plos One Global Public Health a revised version of the manuscript now titled “ Association of lifetime e-cigarette and/or tobacco use and last year cannabis use among university students: a cross-sectional secondary analysis of a National Survey” this paper was coauthored by Lucero Sangster-Carrasco and Dora Blitchtein.

The present document is the letter that includes the “Response to Reviewers” and that addresses each point raised during the review process by the editor and three reviewers,

Thank you for your consideration. We look forward to hearing from you.

Sincerely,

Coautors

Lucero Sanster-Carrasco and	Dora Blitchtein*

*Correspondig author

Universidad Peruana de Ciencias Aplicadas]

Los Nogales 765 Apto 402 San Isidro. Lima, Perú.

+51 999090917

dblit2007@gmail.com

Journal Requirements:

**Please review your reference list to ensure that it is complete and correct. If you have cited papers that have been retracted, please include the rationale for doing so in the manuscript text, or remove these references and replace them with relevant current references. Any changes to the reference list should be mentioned in the rebuttal letter that accompanies your revised manuscript. If you need to cite a retracted article, indicate the article’s retracted status in the References list and also include a citation and full reference for the retraction notice**.

Comment addressed; we included an updated scientific paper reference instead of the book bibliographic reference previously included.

Takahashi A, Kurosawa T. Regression correlation coefficient for a Poisson regression model. Comput Stat Data Anal. 2016;98:71-8. instead of Dohoo IR, Martin W, Stryhn HE. Veterinary epidemiologic research. Charlottetown, P.E.I University of Prince Edward Island; 2009.

Reviewers' comments:

Reviewer's Responses to Questions

**Comments to the Author**

1. If the authors have adequately addressed your comments raised in a previous round of review and you feel that this manuscript is now acceptable for publication, you may indicate that here to bypass the “Comments to the Author” section, enter your conflict of interest statement in the “Confidential to Editor” section, and submit your "Accept" recommendation.

Reviewer #1: All comments have been addressed

Reviewer #2: All comments have been addressed

Reviewer #3: (No Response)

2. Does this manuscript meet PLOS Global Public Health’s [publication criteria](https://journals.plos.org/globalpublichealth/s/criteria-for-publication)? Is the manuscript technically sound, and do the data support the conclusions? The manuscript must describe methodologically and ethically rigorous research with conclusions that are appropriately drawn based on the data presented.

Reviewer #1: Yes

Reviewer #2: Partly

Reviewer #3: Partly

3. Has the statistical analysis been performed appropriately and rigorously?

Reviewer #1: Yes

Reviewer #2: Yes

Reviewer #3: Yes

4. Have the authors made all data underlying the findings in their manuscript fully available (please refer to the Data Availability Statement at the start of the manuscript PDF file)?

The [PLOS Data policy](https://journals.plos.org/globalpublichealth/s/data-availability) requires authors to make all data underlying the findings described in their manuscript fully available without restriction, with rare exception. The data should be provided as part of the manuscript or its supporting information, or deposited to a public repository. For example, in addition to summary statistics, the data points behind means, medians and variance measures should be available. If there are restrictions on publicly sharing data—e.g. participant privacy or use of data from a third party—those must be specified.

Reviewer #1: Yes

Reviewer #2: Yes

Reviewer #3: Yes

5. Is the manuscript presented in an intelligible fashion and written in standard English?

PLOS Global Public Health does not copyedit accepted manuscripts, so the language in submitted articles must be clear, correct, and unambiguous. Any typographical or grammatical errors should be corrected at revision, so please note any specific errors here.

Reviewer #1: Yes

Reviewer #2: No

Reviewer #3: No

**B. Answers to reviewers’ comments**

**Review Comments to the Author**

Reviewer #1:

**There was coherence in comments by authours on common themes raised across different reviewer and subsequent implementation in the manuscript. Corrections and consideration on presenation of tables has been rigorous**.

**1. There is also a clear desire to stick with original hypothesis/theory i.e. on predictable progression therory (Gateway theory) in drug use. The data deficiencies around this theory have however been outlined with the revised submission**

**Comment addressed**

We reviewed this and other alternative mechanisms. We have updated this information so that we are not fixed with only Gateway theory , they can be presented in combination. (lines 585 to 869)

Reviewer #2:

Most of my comments are addressed.

2a. **“However, I still need the abstracts to be re-written. For example one can't understand what you are saying in the opening sentence in the abstract’.**

Comment accepted, Suggestion was included in the abstract (lines 27-29).

2b**. “Similarly, conclusion section conclude the research and provide policy implications and future direction to research on this topic and NOT the summary of results”.**

Comment accepted, Suggestion was included in the conclusion section (2016-2022)

2c. **“Finally, I would add Correlates or Determinants or Factors as well as Peru in the title of the paper e.g. "Correlates of Lifetime e-cigarette and/or tobacco use and last year cannabis use among Peruvian university students: a cross sectional secondary analysis of a National Survey".**

Comment addressed we changed the title “Association of lifetime e-cigarette and/or tobacco use and last year cannabis use among university students: a cross-sectional secondary analysis of a National Survey.”

**2d.Finally expand any abbreviations used in the Abstract (PRa or CI95, etc.).**

Comment addressed

Suggestion included (lines 36- 38) and was reviewed in all the manuscript also the english writing of the manuscript has been meticulously revised.

Reviewer #3: Thank you for tackling most of my suggestions.

3. **I firmly believe you should drop all the theoretical aspects described in the introduction and then in the discussion. In both cases the writing is poor because the connection between theoretical ideas is not at all there. When one reads it one has the impression that authors had to for certain reason bring in some "theories" As I said in my first review the manuscript stands descriptively. Descriptive papers are also extremely valuable, particularly after the adjustment, as you did in this version, is properly justified.**

Partially addressed

The english writing of the manuscript has been meticulously revised. The suggestion about dropping the theoretical aspects contrast with the other reviewers’ suggestions. We decided to include them as the other two reviewers suggested. Also we addressed your previous suggestions and improved the discussion (lines 1694 – 1706)

7. PLOS authors have the option to publish the peer review history of their article ([what does this mean?](https://journals.plos.org/globalpublichealth/s/editorial-and-peer-review-process#loc-peer-review-history)). If published, this will include your full peer review and any attached files.
